# Supplementary material for: Investigating the influence of physiologically relevant hydrostatic pressure on CHO cell batch culture
Source: Sci Rep. 2021 Jan 8;11:162. doi: 10.1038/s41598-020-80576-8 (PMC7794228; doi:10.1038/s41598-020-80576-8)
Supplement: Supplementary file 1 — Supplementary Information 1. [file 41598_2020_80576_MOESM1_ESM.zip › S1/IgG/Feb14 0 30 60 90/30mmHg Jan16.pdf]

===

Acq. Operator : SYSTEM   Seq. Line : 2
Sample Operator : SYSTEM
Acq. Instrument : HPLC-0039   Location : 2
Injection Date : 2/14/2019 10:58:46 AM   Inj : 1
Inj Volume : 100.000 µl
Method : C:\Users\Public\Documents\ChemStation\1\Data\TK_CHO_IgG 2019-02-14 10-52-34 \CHO_IgG_LC.M (Sequence Method)
Last changed : 2/8/2019 3:17:23 PM by SYSTEM

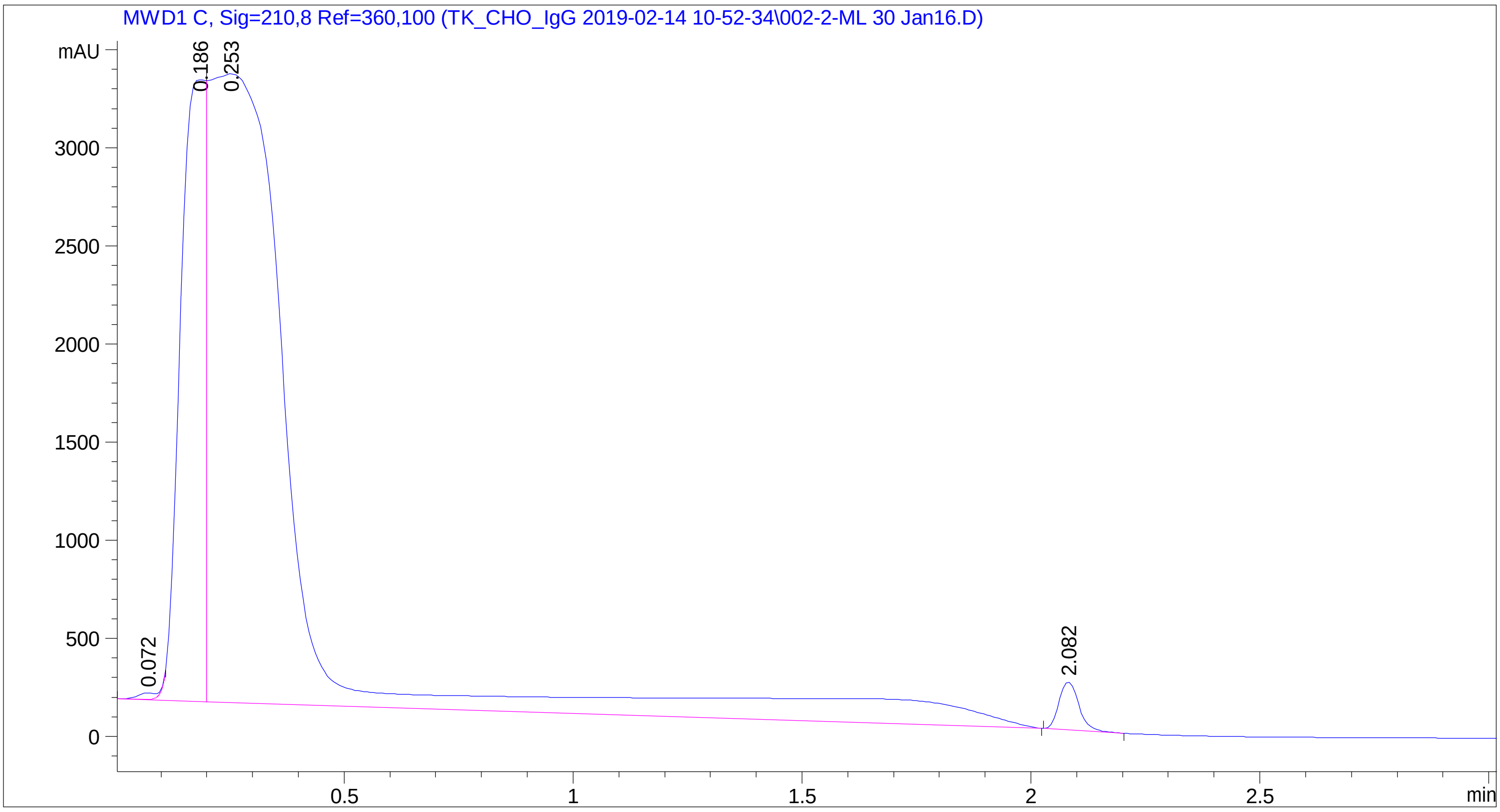

===

Area Percent Report

===

Sorted By : Signal
Multiplier : 1.0000
Dilution : 1.0000
Use Multiplier & Dilution Factor with ISTDs

Signal 1: MWD1 C, Sig=210,8 Ref=360,100

Peak #	RetTime [min]	Type	Width [min]	Area [mAU*s]	Height [mAU]	Area %
1	0.072	BV E	0.0397	93.89975	34.53076	0.1737
2	0.186	VV R	0.0602	1.18117e4	3169.21533	21.8460
3	0.253	VB	0.1888	4.14292e4	3204.67236	76.6242
4	2.082	BBA	0.0471	733.21832	243.64769	1.3561

Totals : 5.40680e4 6652.06615

===

*** End of Report ***
